# Coronary access following ACURATE neo implantation for transcatheter aortic valve-in-valve implantation: *Ex vivo* analysis in patient-specific anatomies

**DOI:** 10.3389/fcvm.2022.902564

**Published:** 2022-09-14

**Authors:** Arif A. Khokhar, Francesco Ponticelli, Adriana Zlahoda-Huzior, Kailash Chandra, Rossella Ruggiero, Marco Toselli, Francesco Gallo, Alberto Cereda, Alessandro Sticchi, Alessandra Laricchia, Damiano Regazzoli, Antonio Mangieri, Bernhard Reimers, Simone Biscaglia, Carlo Tumscitz, Gianluca Campo, Ghada W. Mikhail, Won-Keun Kim, Antonio Colombo, Dariusz Dudek, Francesco Giannini

**Affiliations:** ^1^Department of Cardiology, Imperial College Healthcare National Health Service (NHS) Trust, London, United Kingdom; ^2^Digital Innovations and Robotics Hub, Kraków, Poland; ^3^Gruppo Villa Maria (GVM) Care and Research, Maria Cecilia Hospital, Cotignola, Italy; ^4^Department of Measurement and Electronics, Akademia Gorniczo-Hutnicza (AGH) University of Science and Technology, Kraków, Poland; ^5^Interventional Cardiology, Department of Cardiothoracic and Vascular Science, Ospedale dell’Angelo, Venice, Italy; ^6^Department of Cardiology, ASST Santi Paolo e Carlo, Milan, Italy; ^7^Invasive Cardiology Unit, Humanitas Research Hospital, Istituto di Ricovero e Cura a Carattere Scientifico (IRCCS), Milan, Italy; ^8^Department of Biomedical Sciences, Humanitas University, Milan, Italy; ^9^Cardiovascular Institute, Azienda Ospedaliero-Universitaria di Ferrara, Cona, Italy; ^10^Department of Cardiology and Cardiac Surgery, Kerckhoff Heart Center, Bad Nauheim, Germany; ^11^Institute of Cardiology, Jagiellonian University Medical College, Kraków, Poland

**Keywords:** transcatheter aortic valve implantation (TAVI), valve-in-valve transcatheter aortic valve implantation, coronary access, ACURATE neo™, aortic stenosis

## Abstract

**Background:**

Coronary access after transcatheter aortic valve implantation (TAVI) with supra-annular self-expandable valves may be challenging or un-feasible. There is little data concerning coronary access following transcatheter aortic valve-in-valve implantation (ViV-TAVI) for degenerated surgical bioprosthesis.

**Aims:**

To evaluate the feasibility and challenge of coronary access after ViV-TAVI with the supra-annular self-expandable ACURATE neo valve.

**Materials and methods:**

Sixteen patients underwent ViV-TAVI with the ACURATE neo valve. Post-procedural computed tomography (CT) was used to create 3D-printed life-sized patient-specific models for bench-testing of coronary cannulation. Primary endpoint was feasibility of diagnostic angiography and PCI. Secondary endpoints included incidence of challenging cannulation for both diagnostic catheters (DC) and guiding catheters (GC). The association between challenging cannulations with aortic and transcatheter/surgical valve geometry was evaluated using pre and post-procedural CT scans.

**Results:**

Diagnostic angiography and PCI were feasible for 97 and 95% of models respectively. All non-feasible procedures occurred in ostia that underwent prophylactic “chimney” stenting. DC cannulation was challenging in 17% of models and was associated with a narrower SoV width (30 vs. 35 mm, *p* < 0.01), STJ width (28 vs. 32 mm, *p* < 0.05) and shorter STJ height (15 vs. 17 mm, *p* < 0.05). GC cannulation was challenging in 23% of models and was associated with narrower STJ width (28 vs. 32 mm, *p* < 0.05), smaller transcatheter-to-coronary distance (5 vs. 9.2 mm, *p* < 0.05) and a worse coronary-commissural overlap angle (14.3° vs. 25.6*^o^*, *p* < 0.01). Advanced techniques to achieve GC cannulation were required in 22/64 (34%) of cases.

**Conclusion:**

In this exploratory bench analysis, diagnostic angiography and PCI was feasible in almost all cases following ViV-TAVI with the ACURATE neo valve. Prophylactic coronary stenting, higher implantation, narrower aortic sinus dimensions and commissural misalignment were associated with an increased challenge of coronary cannulation.

## Graphical Abstract

Central illustration: Evaluating feasibility and challenge of coronary cannulation after ViV-TAVI with ACURATE neo valve. 
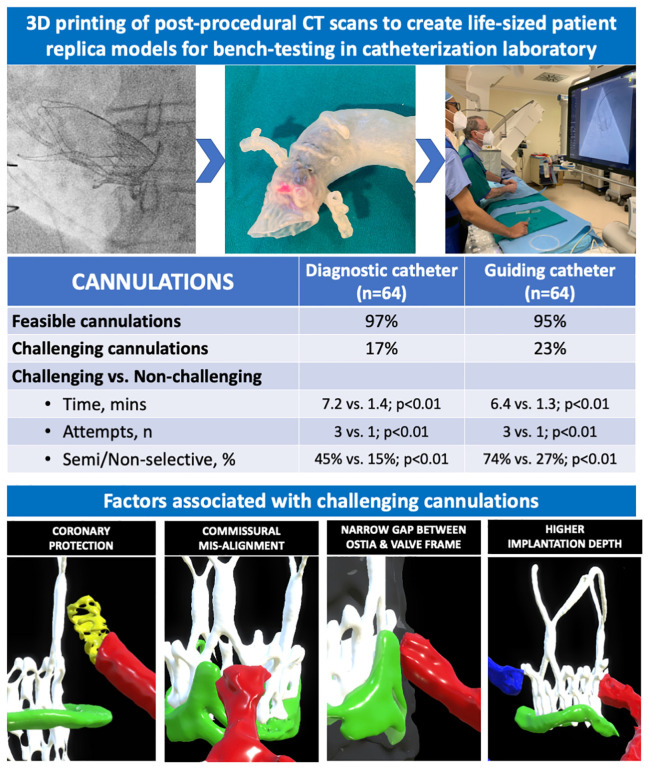

## Introduction

Transcatheter aortic valve-in-valve implantation (ViV-TAVI) is a recommended treatment for degenerated surgical bioprosthetic valves (SBV) in patients deemed high-risk for re-do surgical aortic valve replacement (SAVR) (1). SBV have limited durability and when they fail, ViV-TAVI has shown favourable procedural and clinical outcomes compared to re-do SAVR (2–4). The number of ViV-TAVI procedures is further expected to increase given the expansion of TAVI toward low surgical-risk patients, in whom ViV-TAVI represents a potential treatment strategy for the lifelong management of severe aortic stenosis (5–8). As a consequence, an increased cumulative risk for repeat invasive angiography or percutaneous coronary intervention (PCI) procedures is expected in the next years (9–11). Therefore, the evaluation of coronary access following ViV-TAVI is increasingly relevant when considering the optimal sequential valve treatment for younger patients (7).

Coronary access following TAVI can be challenging and if un-feasible is associated with adverse outcomes (11–13). Prior studies have identified various anatomical, procedural and device-related factors, which can influence the challenge of coronary access following TAVI and TAVI-in-TAVI procedures (14–21). However, comparatively little data exists on coronary access following ViV-TAVI, where the additional presence of the surgical valve frame and leaflets might make coronary re-engagement more challenging (22, 23).

Therefore, we simulated diagnostic angiography and PCI procedures to determine the feasibility and challenge of coronary access in 3D printed patient-specific models derived from a cohort of patients who underwent ViV-TAVI.

## Materials and methods

### Patient cohort

The bench-models were derived from a real cohort of consecutive patients who underwent ACURATE neo (Boston Scientific, Marlborough, MA, USA) valve implantation to treat degenerated SBV across three high-volume European centres between February 2018 and February 2020. All patients were deemed high-surgical risk for re-do SAVR following local heart team discussion. All procedures were performed from transfemoral access and choice of valve sizing and implantation technique was left to the operator’s discretion. Ethical approval for this study was obtained in accordance to the local policy of each institution.

### Imaging analysis

All patients underwent pre and post-procedural contrast-enhanced CT scans using a 128-slice or greater multidetector-row scanner with ECG gating of both systolic and diastolic phases with varying temporal windows to optimise image quality. All images were analysed by three independent cardiologists using a dedicated CT analysis software (Horos, version 3.3.6, OsiriX, Switzerland).

On the pre-procedural scan, baseline measurements of the aortic root, coronaries and surgical bioprosthesis were performed in accordance with current recommendations (24). On the post-procedural scan, the geometrical relationships between the transcatheter valve with the SBV and native aortic/coronary anatomy were evaluated by measuring the vertical and horizontal distances between the coronary ostia and transcatheter valve frame, the implantation depth and the extent of overlap between the coronary ostia and commissural posts (Supplementary Figure 1).

### Creation of 3D printed models

Raw data from each scan was exported in the Digital Imaging and Communication in Medicine (DICOM) format and the aorta, left ventricular blood pool, surgical/transcatheter valves and coronary arteries were segmented using semi-automatic segmentation algorithms (region growing/thresholding/level-tracing) with added manual corrections.

The segmented models were converted into 3D digital models, which were exported as.*stl* files into a computer assisted design (CAD) software (GrabCAD, Stratasys, USA) for 3D printing. Polyjet technology was used to print the patient-specific 3D models (J720 3D printer, Stratasys) (Supplementary Figure 2). The entire aortic arch, ascending aorta and aortic root along with the coronary arteries was printed together using the same material, whilst the surgical and transcatheter heart valves were printed during the same process but using a different more rigid material.

**FIGURE 1:**
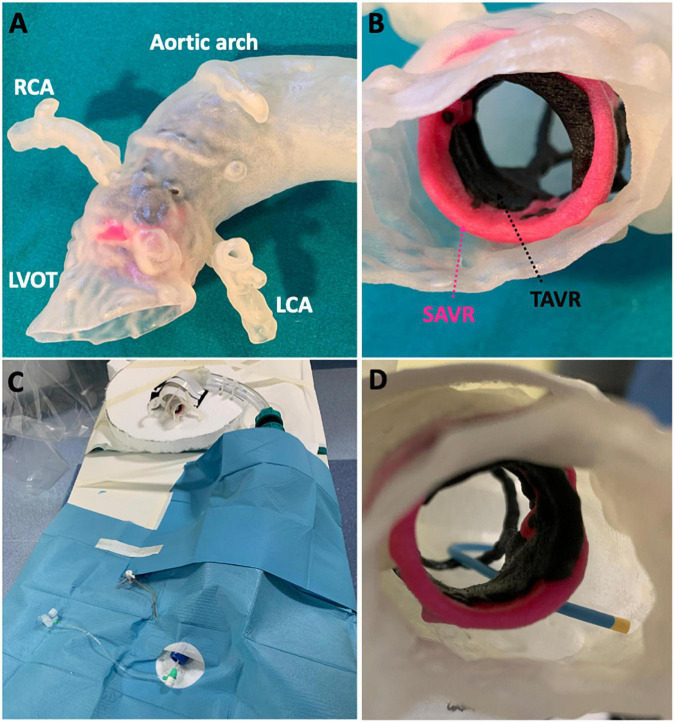
Patient-specific 3D printed models. Post-procedural CT was used to **(A)** 3D print a patient-specific anatomical model complete with **(B)** surgical and transcatheter valves. Each patient model was **(C)** assembled in the catheterization laboratory to simulate **(D)** coronary cannulation procedures.

Each 3D printed model was an exact 1:1 sized replica of the patient’s true anatomy and surgical/transcatheter valve geometry (Figures 1A,B). The materials were selected following preliminary bench-testing to ensure that catheter, wire and device movements closely matched *in vivo* conditions. Each patient model was attached to a prosthetic descending aorta with a femoral access sheath inserted for bench-testing (Figures 1C,D). The authenticity of the final assembled bench-model in terms of performing angiography and PCI procedures was independently confirmed by expert interventional operators (CT, AC, FG, DD).

### Bench-testing

To evaluate the feasibility and challenge of coronary access, each patient-specific bench-model was assembled under cardiac catheterization laboratory conditions, using real equipment to simulate diagnostic angiography and PCI procedures under fluoroscopy (Supplementary Figure 2). Two expert (defined as > 2,000 PCI, 400 TAVI and > 50 ViV-TAVI procedures) interventional cardiologists were instructed to perform a diagnostic angiogram and PCI to both the right and left coronary arteries of each bench-model. Both operators were blinded to the pre and post-procedural CT scan data and each operator was blinded to the cannulation strategies and techniques used by the other operator.

All procedures were performed from the femoral access route using 6Fr catheters. For the diagnostic angiography, the operators were instructed to start with the Judkins right (JR4) and Judkins left (JL4) catheters to reflect conventional practice. If initial cannulation was unsuccessful, the subsequent choice of catheter or cannulation strategy was left to the discretion of the operator. Following diagnostic angiography, the operators were instructed to perform PCI of the proximal right coronary artery (RCA) and left anterior descending artery (LAD). The operators were free to select their preferred guiding catheters, wires and any additional equipment to complete the procedure.

For each procedure, the following data were recorded: cannulation selectivity (selective, semi-selective, non-selective), number of cannulation attempts, fluoroscopy time, catheters used, cannulation techniques and additional equipment used (e.g., guide-extension catheters). The topography of cannulation in relation to the transcatheter heart valve was noted and recorded as either above or below the upper crown and catheter passage inside or outside valve frame.

### Study outcomes

The primary outcome of the study was the feasibility of diagnostic angiography and PCI. A diagnostic angiogram was considered feasible if complete opacification of the coronary vessels was obtained following contrast media injection. A PCI was considered feasible if the vessel was successfully wired distally, a 2.5 mm × 20 mm semi-compliant balloon (Euphora, Boston Scientific, USA) was inflated, followed by successful advancement and retraction of a 3.5 mm × 20 mm drug-eluting stent (Resolute Onyx, Medtronic) in the proximal segment of the vessel.

The secondary outcome was to determine the incidence of challenging diagnostic catheter (DC) and guiding catheter (GC) cannulation, defined as, if for at least one operator either of the two criteria were met:

1. Cannulation was not feasible or non-selective;
2. Cannulation was selective/semi-selective but required greater than 5 min of fluoroscopy time and at least two attempts.

In addition, the association between challenging cannulations with the imaging variables derived from the pre and post-procedural CT scans was evaluated.

### Statistical analysis

Categorical variables are expressed as numbers and percentages and continuous variables as median and interquartile range (IQR) or mean and standard deviation (SD). Categorical data were compared using the chi-squared test or Fisher exact test as appropriate. For comparison of continuous variables, the Student’s *t*-test or Mann-Whitney U test was applied depending on the normality of distribution (assessed by the Kolmogorov-Smirnov test). A 2-sided *p*-value < 0.05 was considered as statistically significant. All statistical analysis were performed using StataIC version 17.0 (StataCorp, IBM).

## Results

Sixteen consecutive patients underwent transfemoral ViV-TAVI with the ACURATE neo valve. Baseline characteristics, procedural and CT imaging data of the study population are presented in Table 1. The mean age was 77 years, 75% were male and mean STS score was 2.09. Mechanism of SBV failure was stenosis, regurgitation or mixed in 6 (38%), 6 (38%) and 4 (25%) cases, respectively. A size S of the ACURATE neo valve was implanted in 13 (81%) cases with prophylactic “chimney” stenting performed for both ostia in 2 (13%) patients. Two operators independently attempted both diagnostic angiography and PCI of each coronary ostium, resulting in a total of 64 diagnostic cannulations and 64 PCI procedures.

**TABLE 1:** Summary of clinical, procedural, and imaging data for each patient in study cohort.

Patient ID	1	2	3	4	5	6	7	8	9	10	11	12	13	14	15	16
**Clinical data**																
Age	79	84	80	81	49	66	82	72	81	71	74	72	75	83	85	63
Sex	M	F	F	M	M	M	M	M	M	M	F	M	M	M	F	M
STS	3.36	3.66	2.6	2.39	0.7	1.27	2.56	1.78	1.71	1.12	2.78	1.32	1.71	2.84	2.91	1.6
**Surgical bioprosthesis**																
Type	MF	MF	P	CE	CE	MF	MF	MF	CE	H	H	P	MF	P	MF	MF
Size	27	23	19	25	27	25	23	25	23	23	25	23	25	23	21	25
Age, years	7	10	17	14	13	9	10	10	17	7	10	2	10	14	10	11
Mechanism of failure	R	S	S	R & S	R	R	S	S	R	R	R & S	R & S	S	R	S	R & S
Aortic Root	Bentall	Normal	Normal	Normal	Hemashield	Normal	Normal	Normal	Normal	Normal	Normal	Normal	Normal	Normal	Bentall	Bentall
**TAVR**																
ACURATE size	M	S	S	M	L	S	S	S	S	S	S	S	S	S	S	S
Coronary protection	Stent	Stent	No	No	No	No	Wire only	Wire only	No	No	No	No	No	No	Wire only	Wire only
**Pre-procedural CT**																
SoV width	36	28	31	35	47	52	35	33	38	32	38	25	40	30	36	31
STJ width	32	25	28	32	46	39	34	30	31	28	36	25	35	28	30	34
STJ height	28	15	11	21	26	32	17	17	21	17	17	9	22	14	31	17
LCA height	5	3.5	3	11	2.5	11	5.5	5.5	16.5	3.2	7.5	5.2	12.5	8	1	6.5
RCA height	5	4	6	14.5	17	21	12	12	17	10	7.6	3	12	11.5	14	8.2
LCA VTC	3.8	3.9	4	10	11	20	10	10	11	9.4	9.6	3	11	5.5	6.17	6
RCA VTC	4.6	7.5	4.5	5	12.3	19.2	8	8	8.1	4	8.8	5	10.5	4.5	7.2	6
**Post-procedural CT**																
Implant depth	9.9	3.8	3.6	4.3	5.52	8.4	6.6	0.55	4.2	4.8	4.9	3.2	5.6	-0.5	1	5.9
LCA MTC	6.2	5	4	14	12.9	20	9.9	5.4	10.7	9.2	12.1	4.9	10.7	6.2	9.7	6.1
RCA MTC	3	4.7	6.9	8.2	15.5	22.5	7	6.2	3.4	1.6	13	5.6	14.6	4.4	9.3	8.1
LCA CCA	19.22	19.5	26.41	17.5	24.16	14.27	2.47	25.46	35.44	11.13	61.14	19.15	63.18	36.45	25.57	67.43
RCA CCA	34	4.17	12.3	41.1	51.6	2.2	5.23	49.34	16.17	5.3	31.16	18.41	30.42	11.46	41.55	36.58

M, male; F, female; STS, society of thoracic surgeons predicted risk of mortality; MF, mitroflow; P, perimount; CE, Carpentier Edwards; H, hancock; R, regurgitation; S, stenosis; TAVR, transcatheter aortic valve replacement; SoV, Sinus of Valsalva; STJ, sinotubular junction; LCA, left coronary artery; RCA, right coronary artery; VTC, virtual transcatheter-to-coronary distance; MTC, measured transcatheter-to-coronary distance; CCA, coronary-commissural angle.

### Diagnostic angiography

**FIGURE 2:**
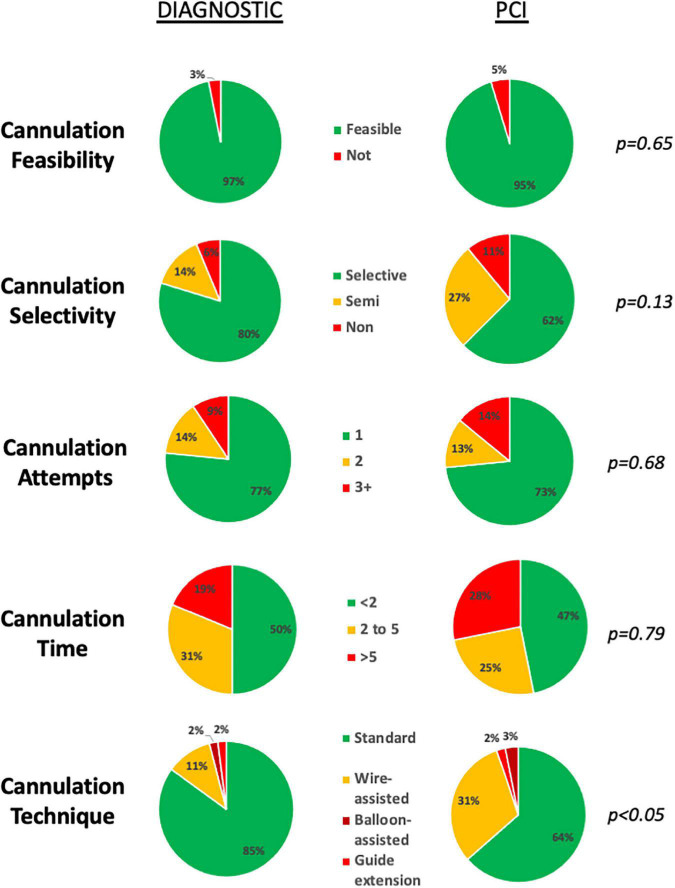
Feasibility and challenge of diagnostic and guiding catheter cannulations. Cannulation feasibility, selectivity, number of attempts and time was equivalent for both diagnostic and guide catheter cannulations. Use of advanced techniques, was more frequently required for guiding catheter cannulations.

The primary outcome of diagnostic angiography feasibility was observed for 62/64 (97%) cannulations. Cannulation was not feasible for both operators in one RCA ostium, which underwent prophylactic stenting during the ViV-TAVI procedure. Data on DC cannulations are presented in Table 2. Median number of cannulation attempts was 1 (IQR: 1-1) and median cannulation time was 1 min 48 s (IQR: 1 min 5 s–3 min 11 s). The majority of cannulations required no more than a single cannulation attempt (49/62, 79%). Selective cannulation was achieved in 51/62 (82%) and most of the cannulations required less than 2 min of fluoroscopy time (32/62, 52%). Advanced cannulation techniques were required for 9/62 (15%) cannulations, with 0.014” coronary wire-assisted cannulation being the main technique of choice (Figure 2). There was no significant difference observed in cannulation feasibility, selectivity, attempts, time or technique used between the LCA and RCA (Table 2).

**TABLE 2:** Data on diagnostic catheter cannulations.

	Both ostia (*n* = 64)	LCA (*n* = 32)	RCA (*n* = 32)	*P*-value
Angiography feasibility	62 (97%)	32 (100%)	30 (94%)	*p* = 0.49
**Cannulation selectivity**				
Selective	51 (82%)	25 (78%)	26 (87%)	*p* = 0.94
Semi-selective	9 (15%)	5 (16%)	4 (13%)	
Non-selective	4 (6%)	2 (6%)	2 (7%)	
**Cannulation attempts**				
1	49 (79%)	23 (72%)	26 (87%)	*p* = 0.55
2	9 (15%)	7 (22%)	2 (7%)	
3+	6 (10%)	2 (6%)	4 (13%)	
**Cannulation time**				
<2 min	32 (52%)	14 (44%)	18 (60%)	*p* = 0.44
2–5 min	20 (32%)	12 (38%)	8 (27%)	
>5 min	12 (19%)	6 (19%)	6 (20%)	
**Cannulation advanced techniques**				
Wire-assisted	7 (11%)	5 (16%)	2 (7%)	*p = 0.46*
Guide-extension	1 (2%)	1 (3%)	0 (0%)	
Balloon-assisted	1 (2%)	1 (3%)	0 (0%)	

Data presented as n (%). LCA, left coronary artery; RCA, right coronary artery.

**FIGURE 3:**
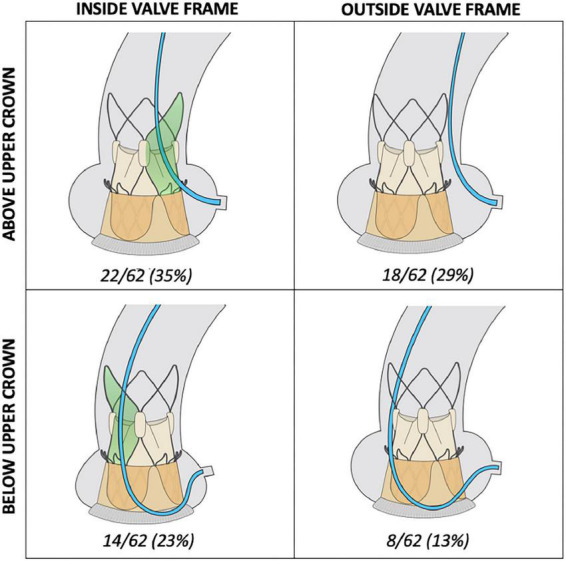
Topography of diagnostic cannulation in relation to valve frame. Access to the coronary ostium was pre-dominantly achieved from above the upper crown of the ACURATE neo valve (64%). In 42% of cannulations, coronary cannulation was successfully performed by completely bypassing the valve frame.

Choice of catheters selected and topography of cannulation in relation to the valve frame is shown in Supplementary Figure 3 and Figure 3, respectively. Differences in procedural outcomes between the operators as well as the individual operator’s perceived level of difficulty for performing diagnostic angiography are reported in Supplementary Table 1 and Supplementary Figure 4, respectively.

### Percutaneous coronary intervention

Performing a complete PCI procedure was feasible in 61/64 (95%) of cases. PCI could not be performed for ostia that underwent prophylactic coronary stenting (1 LCA cannulation, 2 RCA cannulations) (Table 3).

**TABLE 3:** Data on guiding catheter cannulations.

	Both ostia (*n* = 64)	LCA (*n* = 32)	RCA (*n* = 32)	*P*-value
Procedural feasibility	61 (95%)	31 (97%)	30 (94%)	*p* = 0.50
Guiding catheter cannulation	61 (95%)	31 (97%)	30 (94%)	*p* = 0.50
Vessel wiring	61 (95%)	31 (97%)	30 (94%)	*p* = 0.50
Vessel POBA	61 (95%)	31 (97%)	30 (94%)	*p* = 0.50
Vessel stenting	61 (95%)	31 (97%)	30 (94%)	*p* = 0.50
**Cannulation selectivity**				
• Selective	40 (66%)	19 (59%)	21 (66%)	*p* = 0.87
• Semi-selective	17 (28%)	9 (28%)	8 (25%)	
• Non-selective	7 (11%)	4 (13%)	3 (9%)	
**Cannulation attempts**				
• 1	47 (77%)	24 (75%)	23 (72%)	*p* = 0.58
• 2	8 (13%)	6 (19%)	2 (6%)	
• 3+	9 (15%)	2 (6%)	7 (22%)	
**Cannulation time**				
• <2 min	30 (49%)	16 (50%)	14 (44%)	*p* = 0.52
• 2–5 min	16 (26%)	9 (28%)	7 (22%)	
• >5 min	18 (30%)	7 (22%)	11 (34%)	
**Cannulation techniques**				
• Standard	39 (64%)	19 (59%)	2 (63%)	*p* = 0.63
• Wire-assisted	19 (31%)	10 (31%)	20 (63%)	
• Guide-extension	2 (3%)	2 (6%)	0 (0%)	
• Balloon-assisted	1 (2%)	0 (0%)	1 (2%)	

Compared to DC, a greater percentage of GC cannulations were either semi- (14 vs. 27%) or non-selective (6 vs. 11%) (Figure 2). Median cannulation time was 2 min 5 s (IQR: 48 s-5 min 12 s). Advanced techniques to achieve guide catheter cannulation were required in 22/64 (34%) of cannulations, with the use of a 0.014” coronary wire only or in addition to a guide-extension catheter or balloon-assisted technique required for 19/64 (30%), 2/64 (3%) and 1/64 (2%) of cannulations, respectively (Table 3). A wider selection of guiding catheters were selected particularly for cannulation of the LCA (Figure 3).

Differences in procedural outcomes between the operators as well as the individual operator’s perceived level of difficulty for performing PCI are reported in Supplementary Table 1 and Supplementary Figure 4, respectively.

### Challenging cannulation

The secondary outcome criteria for challenging cannulation were met for 11/64 (17%) of DC and 15/64 (23%) of GC cannulations. Data regarding the cannulation procedures, transcatheter and surgical valve types and pre- and post-CT imaging analysis for challenging DC and GC cannulations is presented in Supplementary Tables 2, 3.

Challenging cannulations were associated with a longer cannulation time (DC: 7 min 16 s vs. 1 min 25 s, *p* < 0.01; GC: 6 min 9 s vs. 1 min 15 s, *p* < 0.01), required a greater number of attempts [DC: 3 (IQR: 2–4) vs. 1 (IQR: 1–1), *p* < 0.01; GC: 3 (IQR: 2–3) vs. 1 (IQR: 1–1), *p* < 0.01] and were associated with more semi- and non-selective cannulations (DC: 45 vs. 15%, *p* < 0.01; GC: 74 vs. 27%, *p* < 0.01) (Table 4).

**TABLE 4:** Differences in procedural and CT imaging data between challenging and non-challenging diagnostic and guiding catheter cannulations.

	Diagnostic catheter cannulation			Guiding catheter cannulation		
	Challenging *n* = 11	Non-challenging *n* = 53	*P*-value	Challenging *n* = 15	Non-challenging *n* = 49	*P*-value
**Cannulation data**						
Cannulation feasibility	9 (82%)	53 (100%)	0.05	12 (80%)	49 (100%)	<0.05
Cannulation time, min	7.16 (5.2–10.23)	1.42 (1.04–2.39)	<0.01	6.35 (5.2−14.1)	1.25 (0.46−2.45)	<0.01
Cannulation attempts	3 (2-4)	1 (1-1)	<0.01	3 (2-3)	1 (1-1)	<0.01
**Cannulation selectivity**						
• Selective	6 (55%)	45 (85%)	<0.01	4 (27%)	36 (73%)	<0.01
• Semi-selective	1 (9%)	8 (15%)		4 (27%)	13 (27%)	
• Non-selective	4 (36%)	0 (0%)		7 (47%)	0 (0%)	
**Cannulation techniques**						
• Standard	4 (36%)	50 (94%)	<0.01	1 (7%)	38 (78%)	<0.01
• Wire-assisted	3 (27%)	3 (6%)		8 (53%)	10 (20%)	
• Balloon-assisted	1 (9%)	0 (0%)		1 (7%)	0 (0%)	
• Guide-extension catheter	1 (9%)	0 (0%)		2 (13%)	1 (2%)	
**Pre-procedural CT**						
Coronary height, mm	6 (4–8)	8.2 (5–12)	0.21	6 (4–10)	8.2 (5–12.5)	0.29
Sinus of Valsalva width, mm	30 (28–31)	35 (32–38)	<0.01	31 (28–36)	35 (32–38)	0.05
Sinotubular junction width, mm	28 (25–34)	32 (30–35)	<0.05	28 (25–32)	32 (30–35)	<0.05
Sinotubular junction height, mm	15 (11–17)	17 (17–26)	<0.05	15 (11–28)	17 (17–22)	0.09
Virtual transcatheter-to-coronary distance, mm	5.5 (4.5–7.5)	7.2 (5–10)	0.32	4.6 (4–7.5)	8 (6–10)	0.07
**Post-procedural CT**						
Implantation depth, mm	3.8 (3.2–5.9)	4.8 (3.6–5.6)	0.23	3.8 (3.6–6.6)	4.8 (3.2–5.6)	0.57
**Relationship to risk plane**						
• Above	2 (18%)	22 (42%)	0.13	3 (20%)	21 (43%)	0.14
• Below	9 (82%)	31 (58%)		12 (80%)	28 (57%)	
Measured transcatheter-to-coronary distance, mm	6.1 (4.9–6.9)	8.2 (5.4–12.1)	0.30	5 (4–6.9)	9.2 (6.1–12.1)	<0.05
Coronary-commissural angle, degrees	18.4 (4.2–36.5)	25.6 (16.2–36.6)	0.19	14.3 (5.3–26.4)	25.6 (17.5–41.1)	<0.01

Values are n (%), mean ± SD or median (IQR).

Use of non-standard cannulation techniques (0.014” coronary guide wire-assisted, guide-extension catheter or balloon-assisted cannulation) were more frequently required for challenging versus non-challenging DC (wire: 27 vs. 6%, guide-extension: 9 vs. 0%, balloon: 9 vs. 0%; *p* < 0.01) and GC cannulations (wire: 53 vs. 20%, guide-extension: 13 vs. 2%, balloon: 7 vs. 0%; *p* < 0.01) (Table 4).

### Factors associated with challenging cannulations

**FIGURE 4:**
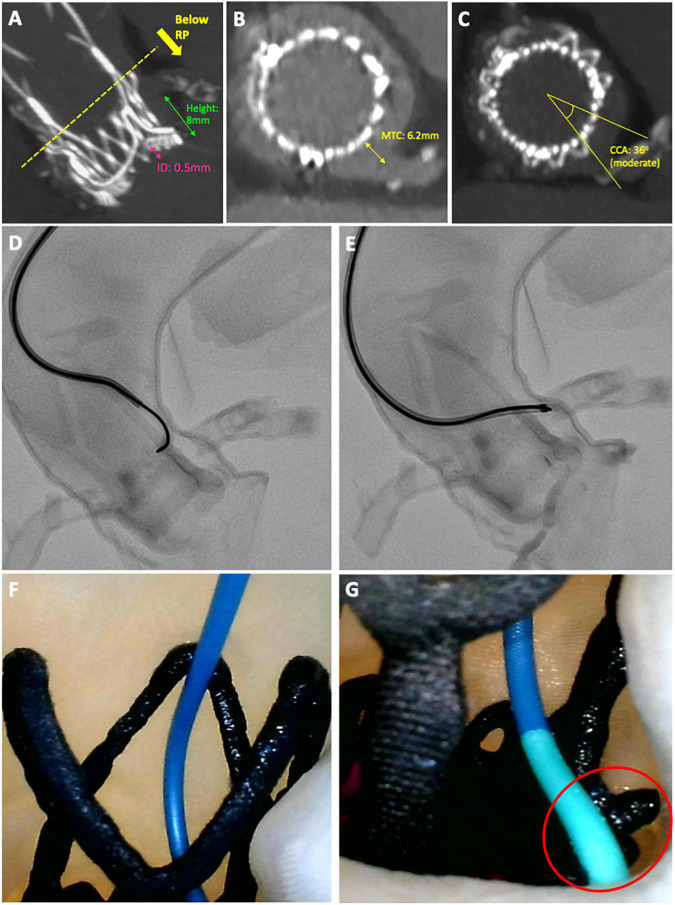
Case example of a challenging diagnostic cannulation. An 83-year old male underwent ACURATE neo implantation to treat a 14-year old degenerated Perimount23 surgical bioprosthesis. Post-procedural CT showed **(A)** a high implantation with low-lying coronary arteries, **(B)** a narrow gap between valve frame and aortic wall and **(C)** moderate overlap between the commissural posts and coronary arteries, all implying challenging cannulation. **(D,E)** Semi-selective cannulation of the LCA was achieved using an Amplatz Left 2 guiding catheter with 0.014 wire-assistance, after 17 min of fluoroscopy time and four attempts. **(F,G)** Camera placed internally demonstrating cannulation technique of approaching the ostium from above and resting the distal tip of the guiding catheter on the upper crown of the ACURATE neo adjacent to the LCA.

Challenging DC and GC occurred when coronary ostia arose below the upper crown of the THV, there was a narrow sinus gap between the transcatheter/surgical valve frames and aortic wall, commissural mis-alignment and when prophylactic “chimney” stenting was performed. In the most challenging cases, the combination of these factors resulted in prolonged cannulation times, requiring multiple attempts with the use of advanced techniques (Figure 4).

Differences in pre- and post-procedural CT imaging data were evaluated between challenging and non-challenging cannulations (Table 4). Aortic sinus dimensions, as evaluated by SoV and STJ width, were smaller for both challenging DC (*p* < 0.05) and GC (*p* = 0.05) cannulations. Challenging cannulations were associated with lower left (3.6 vs. 4.8 mm) and right (3.8 vs. 4.8 mm) coronary heights but this difference was not statistically significant.

On post-procedural CT, the measured transcatheter-to-coronary (MTC) distance (5 vs. 9.2 mm; *p* < 0.05) and commissure-coronary angle (14.3° vs. 25.6°; *p* < 0.01) were both significantly lower for challenging GC cannulations. Implantation depth was numerically lower (DC: 3.8 vs. 4.8 mm, *p* = 0.23; GC: 3.8 vs. 4.8 mm; *p* = 0.57) for challenging cannulations.

**FIGURE 5:**
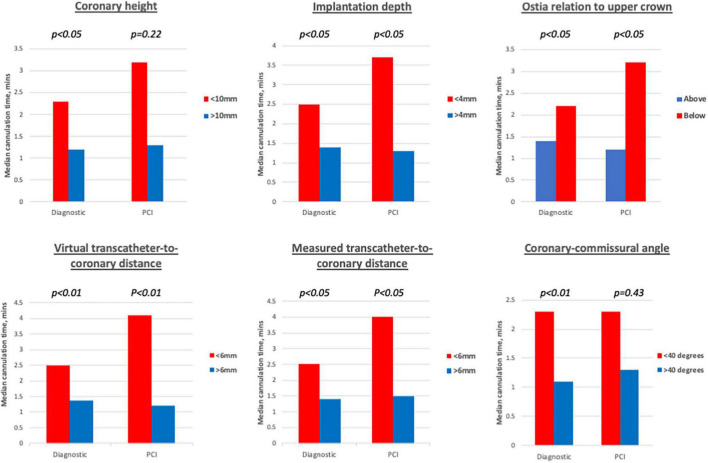
Diagnostic cannulations times associated with different imaging cut-offs. Prolonged diagnostic cannulation times were observed when coronary height < 10 mm, implantation < 4 mm, ostia arose below the upper crown, virtual and measured transcatheter-to-coronary distances < 6 mm and coronary-commissural angle < 40 degrees. For guiding catheter cannulations, significantly prolonged cannulation times were only observed for implantation depth < 4 mm, ostia arising below the upper crown, virtual, and measured transcatheter-to-coronary distances < 6 mm.

The impact of each pre- and post-CT imaging parameter on diagnostic and guiding catheter cannulation time, numbers of attempts and selectivity are presented in Supplementary Tables 4, 5. In summary, cannulation time with both DC and GC was prolonged when either the implantation depth was < 4 mm, coronary ostia were located below the upper crown or the virtual-transcatheter distance (VTC) was < 6 mm (*p*-values for all < 0.05) (Figure 5). For DC cannulation, an implantation depth < 4 mm, was associated with increased cannulation attempts and worsening cannulation selectivity. For GC cannulations increased attempts and worsening cannulation selectivity was observed when the MTC < 6 mm and the coronary-commissural angle was < 40° (*p*-values for all < 0.05).

## Discussion

This exploratory study is the first study to systematically evaluate diagnostic and guide catheter cannulation following ViV-TAVI with the ACURATE neo valve. Bench-testing of patient-specific 3D printed models demonstrated (Graphical Abstract):

1) Feasibility to perform diagnostic angiography and PCI was 97 and 95%, respectively.
2) Seventeen percentage of diagnostic and 23% of guiding catheter cannulations were challenging requiring greater fluoroscopy time, number of attempts, semi- or non-selective cannulation and use of advanced techniques.
3) The main reasons for challenging cannulation were prophylactic stenting performed during the ViV-TAVI procedure, smaller aortic sinus dimensions, severe commissural mis-alignment and when ostia arose below the upper crown of the valve, which could be due to higher implantation depths or lower coronary heights.
4) Implantation depth < 4 mm, virtual transcatheter-to-coronary (VTC) distance < 6 mm, measured transcatheter-to-coronary (MTC) distance < 6 mm and coronary-commissural angle < 40° were more frequently observed in challenging cannulations.

Assessing the feasibility of coronary access and PCI is increasingly relevant as TAVI expands toward younger and lower-risk patients who have an increased life-time risk for repeat invasive angiography due to progression of CAD (9–12). Studies using pre or post-procedural CT to virtually assess coronary access have suggested that challenging or un-feasible cannulation may occur following 9–35% TAVR (16–18), 27–78% of TAVI-in-TAVI (19, 20, 25) and 58% of ViV-TAVI (22) procedures. However, data from real studies of post TAVI cannulation are more re-assuring with success rates for diagnostic cannulation and PCI success ranging between 90–100 and 92–97%, respectively, even in the acute setting (11–14, 26–28).

In our study the feasibility for diagnostic angiography and PCI was 97 and 95%, respectively. These findings are encouraging in the setting of ViV-TAVI, where the presence of the SBV frame and leaflets should further hamper coronary cannulation. Several explanations for these favourable results could be considered. All cannulations were performed by senior interventionists experienced in catheter selection and cannulation techniques required for post-TAVI coronary access (27). Moreover, the transcatheter valve design may have a significant impact upon the feasibility of coronary access (14, 16). All the patients underwent ViV-TAVI with the ACURATE neo valve, which has a split-level design consisting of a short lower stent frame and large open-celled upper stabilisation arches. This unique design is potentially advantageous for coronary access, as it provides operators with different possible cannulation routes to the coronary ostium (Figure 3). In contrast, valves with larger stent-frames such as the Corevalve/Evolut platform are associated with more challenging or un-feasible coronary cannulation although data is conflicting (11–14).

### Factors associated with challenging cannulation

A combination of multiple anatomical, procedural and device-related factors contribute to the challenge of coronary access (14, 27). Proposed classification schemes for coronary access have highlighted three key factors: (1) implantation depth, (2) gap between valve frame and aortic wall and the (3) extent of commissural alignment (15, 29). Previously we showed that an implantation depth > 4 mm suggested more favourable coronary access without impacting upon post-procedural gradients (22). Similarly, our bench study demonstrates that an implantation depth > 4 mm was associated with a shorter cannulation time, more selective cannulations and fewer cannulation attempts when using a DC. Moreover, GC cannulation times were also shorter with advanced techniques required less frequently. The reason for favourable coronary access at lower valve implantations is that more ostia are likely to be located above the covered stent frame, which cannot be traversed by a catheter.

Ostia deemed challenging to cannulate had smaller aortic dimensions as assessed by SoV and STJ width. A narrow aortic sinus translates into a smaller gap available between the THV frame and aortic wall for subsequent catheter entry. Previous studies have used different methodologies to measure and evaluate this gap (14, 16–19, 22, 30), with current classification schemes suggesting that a 2 mm cut-off distance identifies challenging or un-feasible coronary access (15, 29). However, in our cohort the median MTC distance was 7.55 mm and only one coronary ostium had a MTC gap < 2 mm. In contrast, we found that a cut-off of 6 mm was a more useful discriminator for challenging cannulation, with prolonged diagnostic cannulation times, increased attempts for guide catheter cannulation and fewer selective cannulations achieved when the MTC was < 6 mm compared to > 6 mm. Although a 2 mm gap allows for a 6Fr (∼1.8 mm) catheter to enter the aortic sinus, it may not account for the additional space required to manoeuvre the catheter in order to achieve stable, supportive and selective cannulation, particularly for guiding catheters. Greater insights into the necessary space required for successful cannulation might be obtained by analyzing the volume and three-dimensional morphology of the aortic sinuses (30).

Overlap between a THV commissural post and coronary ostia is common following TAVR and may pose a significant challenge to coronary cannulation (16, 18, 29, 31). However, to date no study has evaluated how the extent of commissural-coronary overlap can directly influence cannulation challenge and feasibility. In our cohort, severe overlap or mis-alignment, defined as a coronary-commissural angle (CCA) < 20° was present in 15/32 (47%) coronary ostia. The average CCA for challenging DC and GC cannulation was 14.3° and 18.4°, respectively. However, the impact of severe overlap was greater for GC cannulations, which required more attempts and advanced techniques and resulted in fewer selective cannulations. This finding could be explained by the fact that guiding catheters are stiffer, and the reduced flexibility makes it more challenging to navigate around the obstacle of the THV commissural post. Therefore, a 0.014” coronary wire or guide-extension catheter is often required to achieve selective cannulation with adequate support to complete the PCI. The incidence of severe coronary overlap is expected to be lower as procedural techniques designed to achieve commissural alignment with the ACURATE neo valve are adopted (32). Of note, in our cohort systematic techniques to achieve commissural alignment with the ACURATE neo valve were not adopted. However, the impact of these techniques in reducing the challenge of coronary access, particularly in the setting of ViV-TAVI remains to be determined.

Coronary protection with the chimney technique resulted in 2/64 (3%) DC and 3/64 (5%) GC cannulations being unfeasible. In one patient, PCI to both coronary arteries was not feasible due to an inability to cannulate the neo-ostia, which were located in an un-favourable position for cannulation (Supplementary Figure 5). This highlights the importance selecting an appropriate coronary protection strategy, which will also maintain long-term coronary access. Consideration should be given to the stent positioning, extent of stent protrusion and for certain cases alternative coronary protection strategies such as Bioprosthetic Aortic Scallop Intentional Laceration Coronary Artery (BASILICA) may be considered (33).

### Limitations

Our study is limited to a small sample of ViV-TAVI patients in whom only the ACURATE neo valve was utilised, therefore these findings cannot be applied to other transcatheter heart valves. Cannulations were performed by two experienced interventional operators and variations in their preferred techniques could have accounted for some of the observed differences. Furthermore, cannulation challenge and feasibility may vary amongst operators particularly due to different levels of expertise in post-TAVI coronary access. However, in this context, the fact that certain cannulations were challenging or even un-feasible for experienced operators is highly relevant. Due to the design and assembly of the bench-models, all cannulations were performed from the femoral access route and catheters and techniques selected could be different if the cannulations were performed from the trans-radial access. The prosthetic descending aorta and femoral access may not have replicated real-life ilio-femoral tortuosity which can contribute to the challenge of coronary cannulation. Finally, the nature of bench-testing means that these results were obtained following cannulation of static ostia in *ex vivo* models, which may not fully reflect the dynamic *in vivo* conditions. However, given that these models were 3D printed based on post-procedural CT scans, ensured that the complex anatomical relationships between the transcatheter/surgical valves with surrounding aorta and coronary ostia was preserved.

## Conclusion

In this exploratory bench-analysis, diagnostic angiography and PCI was found to be highly feasible following ViV-TAVI with the ACURATE neo valve. Important factors associated with non-feasible or challenging cannulation included prophylactic “chimney” stenting, higher implantation, narrower aortic sinus dimensions and severe commissural misalignment.

## Data availability statement

The original contributions presented in this study are included in the article/Supplementary material, further inquiries can be directed to the corresponding author.

## Ethics statement

The studies involving human participants were reviewed and approved by Maria Cecilia Hospital, Cotignola, Italy. The patients/participants provided their written informed consent to participate in this study. Written Informed Consent was obtained for the publication of any potentially identifiable images or data included in this article.

## Author contributions

AK, FP, AZ-H, CT, ACo, DD, and FGa contributed to conception and design of the study. AK, ACo, MT, AL, RR, and KC collected the data and maintained the database for the duration of the study. AK, FGi, AL, and FGa performed the computed tomography analysis and maintained the imaging database for the study. AK, AS, SB, and GC performed statistical analysis. AK wrote the first draft of the manuscript. AZ-H, AS, W-KK, GM, and FGi wrote sections of the manuscript. All authors contributed to manuscript revision, read, and approved the submitted version.

## Supplementary material

Supplementary Figure 1 Post-procedural CT analysis. For each coronary ostium in the cohort, post-procedural CT analysis of implantation depth, relationship to upper crown, gap between valve frame and ostium and coronary-commissural angle was performed.

Supplementary Figure 2 Creation of 3D-printed model. Study flowchart demonstrating how post-procedural CT was segmented and processed to print the 3D models, which were then assembled for bench-testing under real catheterization laboratory conditions.

Supplementary Figure 3 Diagnostic and guiding catheters selected for cannulation. The Judkins Right 4 catheter was the most frequently selected catheter for the RCA whilst a greater range of catheters diagnostic and particularly guiding catheters were selected for cannulating the LCA.

Supplementary Figure 4 Operator reported difficulty in diagnostic and PCI cannulation for each patient model. The two operators were asked to report their difficulty in completing the diagnostic and PCI cannulations for each ostium. Responses were recorded on a scale of 1-10 (1 = extremely easy).

Supplementary Figure 5 Location of neo-ostium following chimney stenting. Case example of an 84-year old female who underwent ACURATE neo implantation to treat a degenerated Mitroflow prosthesis. Due to high-risk for coronary obstruction, coronary protection of the LCA **(A,C)** and RCA **(B,D)** using the “chimney” technique was performed. The yellow start denotes the location of the neo-stent which was un-feasible to cannulate.

## Abbreviations

ViV-TAVI: valve-in-valve transcatheter aortic valve implantation
SAVR: surgical aortic valve replacement
PCI: percutaneous coronary intervention
LCA: left coronary artery
RCA: right coronary artery
DC: diagnostic catheter
GC: guiding catheter
CT: computed tomography
VTC: virtual transcatheter-to-coronary distance
MTC: measured transcatheter-to-coronary distance.

## References

[1] Nishimura RA Otto CM Bonow RO Carabello BA Erwin JP Fleisher LA 2017 AHA/ACC focused update of the 2014 AHA/ACC guideline for the management of patients with valvular heart disease: a report of the American college of cardiology/American heart association task force on clinical practice guidelines. *J Am Coll Cardiol.* (2017) 70:252–89. 10.1016/j.jacc.2017.03.011 28315732

[2] Tam DY Dharma C Rocha RV Ouzounian M Wijeysundera HC Austin PC Transcatheter ViV versus redo surgical AVR for the management of failed biological prosthesis: early and late outcomes in a propensity-matched cohort. *JACC Cardiovasc Interv.* (2020) 13:765–74. 10.1016/j.jcin.2019.10.030 31954671

[3] Bleiziffer S Simonato M Webb JG Rodés-Cabau J Pibarot P Kornowski R Long-term outcomes after transcatheter aortic valve implantation in failed bioprosthetic valves. *Eur Heart J.* (2020) 41:2731–42. 10.1093/eurheartj/ehaa544 32592401

[4] Deharo P Bisson A Herbert J Lacour T Etienne CS Porto A Transcatheter valve-in-valve aortic valve replacement as an alternative to surgical re-replacement. *J Am Coll Cardiol.* (2020) 76:489–99. 10.1016/j.jacc.2020.06.010 32731926

[5] Popma JJ Deeb GM Yakubov SJ Mumtaz M Gada H O’Hair D Transcatheter aortic-valve replacement with a self-expanding valve in low-risk patients. *N Engl J Med.* (2019) 380:1706–15. 10.1056/NEJMoa1816885 30883053

[6] Mack MJ Leon MB Thourani VH Makkar R Kodali SK Russo M Transcatheter aortic-valve replacement with a balloon-expandable valve in low-risk patients. *N Engl J Med.* (2019) 380:1695–705. 10.1056/NEJMoa1814052 30883058

[7] Yerasi C Rogers T Forrestal BJ Case BC Khan JM Ben-Dor I Transcatheter versus surgical aortic valve replacement in young. Low-Risk Patients With Severe Aortic Stenosis. *JACC Cardiovasc Interv.* (2021) 14:1169–80. 10.1016/j.jcin.2021.03.058 34112453

[8] Landes U Sathananthan J Witberg G De Backer O Sondergaard L Abdel-Wahab M Transcatheter replacement of transcatheter versus surgically implanted aortic valve bioprostheses. *J Am Coll Cardiol.* (2021) 77:1–14. 10.1016/j.jacc.2020.10.053 33413929

[9] Vilalta V Asmarats L Ferreira-Neto AN Maes F de Freitas Campos Guimarães L Couture T Incidence, clinical characteristics, and impact of acute coronary syndrome following transcatheter aortic valve replacement. *JACC Cardiovasc Interv.* (2018) 11:2523–33. 10.1016/j.jcin.2018.09.001 30573061

[10] Mentias A Desai MY Saad M Horwitz PA Rossen JD Panaich S Incidence and outcomes of acute coronary syndrome after transcatheter aortic valve replacement. *JACC Cardiovasc Interv.* (2020) 13:938–50. 10.1016/j.jcin.2019.11.027 32061612 PMC7202131

[11] Stefanini GG Cerrato E Pivato CA Joner M Testa L Rheude T Unplanned percutaneous coronary revascularization after TAVR: a multicenter international registry. *JACC Cardiovasc Interv.* (2021) 14:198–207. 10.1016/j.jcin.2020.10.031 33478637

[12] Faroux L Lhermusier T Vincent F Nombela-Franco L Tchétché D Barbanti M ST-Segment elevation myocardial infarction following transcatheter aortic valve replacement. *J Am Coll Cardiol.* (2021) 77:2187–99. 10.1016/j.jacc.2021.03.014 33926655

[13] Kim WK Pellegrini C Ludwig S Möllmann H Leuschner F Makkar R Feasibility of coronary access in patients with acute coronary syndrome and previous TAVR. *JACC Cardiovasc Interv.* (2021) 14:1578–90. 10.1016/j.jcin.2021.05.007 34294400

[14] Barbanti M Costa G Picci A Criscione E Reddavid C Valvo R Coronary cannulation after transcatheter aortic valve replacement. *JACC Cardiovasc Interv.* (2020) 13:2542–55. 10.1016/j.jcin.2020.07.006 33069648

[15] Tarantini G Fabris T Nai Fovino L. TAVR-in-TAVR and coronary access: the importance of pre-procedural planning. *Eurointervention.* (2020) 16:e129–32. 10.4244/EIJ-D-19-01094 32091405

[16] Ochiai T Chakravarty T Yoon SH Kaewkes D Flint N Patel V Coronary access after TAVR. *JACC Cardiovasc Interv.* (2020) 13:693–705. 10.1016/j.jcin.2020.01.216 32192689

[17] Rogers T Greenspun BC Weissman G Torguson R Craig P Shults C Feasibility of coronary access and aortic valve reintervention in low-risk TAVR patients. *JACC Cardiovasc Interv.* (2020) 13:726–35. 10.1016/j.jcin.2020.01.202 32192693

[18] Abdelghani M Landt M Traboulsi H Becker B Richardt G. Coronary access after TAVR with a self-expanding bioprosthesis: insights from computed tomography. *JACC Cardiovasc Interv.* (2020) 13:709–22. 10.1016/j.jcin.2020.01.229 32192691

[19] De Backer O Landes U Fuchs A Yoon SH Mathiassen ON Sedaghat A Coronary access after TAVR-in-TAVR as evaluated by multidetector computed tomography. *JACC Cardiovasc Interv.* (2020) 13:2528–38. 10.1016/j.jcin.2020.06.016 33153567

[20] Buzzatti N Montorfano M Romano V De Backer O Søndergaard L Rosseel L A computed tomography study of coronary access and coronary obstruction after redo transcatheter aortic valve implantation. *Eurointervention.* (2020) 16:e1005–13. 10.4244/EIJ-D-20-00475 32928715

[21] Tarantini G Nai Fovino L Scotti A Massussi M Cardaioli F Rodinò G Coronary access after transcatheter aortic valve replacement with commissural alignment: the ALIGN-ACCESS study. *Circ Cardiovasc Interv.* (2022) 15:e011045. 10.1161/CIRCINTERVENTIONS.121.011045 35167332

[22] Khokhar AA Laricchia A Ponticelli F Kim WK Gallo F Regazzoli D Computed tomography analysis of coronary ostia location following valve-in-valve transcatheter aortic valve replacement with the ACURATE neo valve: implications for coronary access. *Catheter Cardiovasc Interv.* (2021) 98:595–604. 10.1002/ccd.29503 33586278

[23] Tarantini G Dvir D Tang GHL. Transcatheter aortic valve implantation in degenerated surgical aortic valves. *Eurointervention.* (2021) 17:709–19. 10.4244/EIJ-D-21-00157 34665140 PMC9725043

[24] Blanke P Soon J Dvir D Park JK Naoum C Kueh SH Computed tomography assessment for transcatheter aortic valve in valve implantation: the vancouver approach to predict anatomical risk for coronary obstruction and other considerations. *J Cardiovasc Comput Tomogr.* (2016) 10:491–9. 10.1016/j.jcct.2016.09.004 27697505

[25] Forrestal BJ Case BC Yerasi C Shea C Torguson R Zhang C Risk of coronary obstruction and feasibility of coronary access after repeat transcatheter aortic valve replacement with the self-expanding evolut valve: a computed tomography simulation study. *Circ Cardiovasc Interv.* (2020) 13:375–83. 10.1161/CIRCINTERVENTIONS.120.009496 33272031

[26] Htun WW Grines C Schreiber T. Feasibility of coronary angiography and percutaneous coronary intervention after transcatheter aortic valve replacement using a Medtronic™ self-expandable bioprosthetic valve. *Catheter Cardiovasc Interv.* (2018) 91:1339–44. 10.1002/ccd.27346 28988450

[27] Yudi MB Sharma SK Tang GHL Kini A. Coronary angiography and percutaneous coronary intervention after transcatheter aortic valve replacement. *J Am Coll Cardiol.* (2018) 71:1360–78. 10.1016/j.jacc.2018.01.057 29566822

[28] Tarantini G Nai Fovino L Le Prince P Darremont O Urena M Bartorelli AL Coronary access and percutaneous coronary intervention up to 3 years after transcatheter aortic valve implantation with a balloon-expandable valve. *Circ Cardiovasc Interv.* (2020) 13:e008972. 10.1161/CIRCINTERVENTIONS.120.008972 32580586 PMC7373469

[29] Tang GHL Zaid S. Coronary re-access after redo TAVI: a proposed classification to simplify evaluation. *Eurointervention.* (2020) 16:e960–2. 10.4244/EIJV16I12A176 33337324

[30] Khokhar AA Ponticelli F Zlahoda-Huzior A Ruggiero R Kim WK Mangieri A A novel 3D imaging approach to evaluate coronary access following ACURATE neo implantation to treat a degenerated surgical bioprosthesis. *Eurointervention.* (2021) 17:1238–9. 10.4244/EIJ-D-21-00634 34338640 PMC9725003

[31] Tang GHL Zaid S Fuchs A Yamabe T Yazdchi F Gupta E Alignment of transcatheter aortic-valve neo-commissures (ALIGN TAVR): impact on final valve orientation and coronary artery overlap. *JACC Cardiovasc Interv.* (2020) 13:1030–42. 10.1016/j.jcin.2020.02.005 32192985

[32] Bieliauskas G Wong I Bajoras V Wang X Kofoed KF De Backer O Patient-Specific implantation technique to obtain neo-commissural alignment with self-expanding transcatheter aortic valves. *JACC Cardiovasc Interv.* (2021) 14:2097–108. 10.1016/j.jcin.2021.06.033 34538602

[33] Khan JM Dvir D Greenbaum AB Babaliaros VC Rogers T Aldea G Transcatheter laceration of aortic leaflets to prevent coronary obstruction during transcatheter aortic valve replacement: concept to first-in-human. *JACC Cardiovasc Interv.* (2018) 11:677–89. 10.1016/j.jcin.2018.01.247 29622147 PMC6309616

