# Supplementary material for: Coronary access following ACURATE neo implantation for transcatheter aortic valve-in-valve implantation: Ex vivo analysis in patient-specific anatomies
Source: Front Cardiovasc Med. 2022 Sep 14;9:902564. doi: 10.3389/fcvm.2022.902564 (PMC9515364; doi:10.3389/fcvm.2022.902564)
Supplement: Supplementary file 1 [file Data_Sheet_1.docx]

**SUPPLEMENTARY TABLES**

**Supplementary table 1: Comparison of diagnostic and guiding catheter cannulation parameters for both operators**

|  | **DIAGNOSTIC** | | | **PCI** | | |
| --- | --- | --- | --- | --- | --- | --- |
|  | Operator 1  n=32 | Operator 2  n=32 | p-value | Operator 1  n=32 | Operator 2  n=32 | p-value |
| **CANNULATION DATA** |  |  |  |  |  |  |
| Cannulation feasibility | 31 (97%) | 31 (98%) | *na* | 30 (94%) | 31 (98%) | p=0.99 |
| Cannulation time, mins | 2.15 (1.10–3.15) | 1.43 (1.08–3.64) | p=0.51 | 3.16 (1.21-5.21) | 1.36 (0.31-4.73) | p<0.05 |
| Cannulation attempts | 1 (1-2) | 1 (1-1) | p=0.16 | 1 (1-2) | 1 (1-1) | p=0.56 |
| Cannulation selectivity   - Selective - Semi-selective - Non-selective | 23 (72%)  6 (19%)  2 (6%) | 28 (88%)  2 (12%)  2 (12%) | p=0.24 | 17 (%)  11 (%)  4 (%) | 23 (%)  6 (3%)  3 (%) | p=0.32 |
| Cannulation techniques   - Standard - Wire-assisted - Balloon-assisted - Guide-extension catheter | 31 (97%)  1 (3%)  0 (0%)  0 (0%) | 24 (75%)  5 (16%)  1 (3%)  1 (3%) | p<0.05 | 20 (67%)  10 (33%)  0 (0%)  0 (0%) | 19 (59%)  9 (28%)  1 (3%)  2 (6%) | p=0.63 |

**Supplementary Table 2: Overview of procedural, valvular and imaging data for each challenging diagnostic catheter cannulation**

|  |  |  | **CANNULATION** | | | | | **VALVE** | | **PRE-PROCEDURE CT** | | | **POST-PROCEDURE CT** | | | |
| --- | --- | --- | --- | --- | --- | --- | --- | --- | --- | --- | --- | --- | --- | --- | --- | --- |
| **Pt** | Artery | Operator | Type | Attempts | Fluoroscopy time  (mins,sec) | Why challenging | Technique used | SAVR | TAVR | Coronary height | SoV width | STJ width | Implant depth | Above or below RP | MTC | Co-Cor angle |
| **2** | LCA | 1 | Non-selective | 1 | 2,06 | Cor protection stent | Standard (JL4) | Mitroflow 23 | ACURATE S | 3.5 | 28 | 25 | 3.8 | Below | 5 | Severe |
|  | RCA | 1 | Not feasible | - | - | Cor protection stent | - |  |  | 4 |  |  |  | Below | 4.7 | Severe |
|  |  | 2 | Not feasible | - | - | Cor protection stent | - |  |  |  |  |  |  |  |  |  |
| **3** | RCA | 2 | Selective | 5 | 23,27 | Small sinus  Commissural post | Balloon-assisted  (IM) | Perimount 19 | ACURATE S | 6 | 31 | 28 | 3.6 | Below | 6.9 | Severe |
| **6** | LCA | 2 | Selective | 2 | 7,16 | Commissural post | Guide-catheter extension (AL2) | Mitroflow 25 | ACURATE S | 11 | 52 | 39 | 8.4 | Above | 20 | Severe |
|  | RCA | 1 | Selective | 2 | 9,33 | Commissural post  Anterior RCA | Standard (AR1) |  |  | 21 |  |  |  | Above | 22.5 | Severe |
| **14** | LCA | 2 | Non-selective | 2 | 7,02 | Small sinus  Commissural post  Pericardial skirt | Wire-assisted (AR1) | Perimount 23 | ACURATE S | 5.2 | 25 | 25 | 3.2 | Below | 4.9 | Severe |
|  | RCA | 2 | Selective | 4 | 10,32 | Small sinus  Commissural post  Pericardial skirt | Standard (AR1) |  |  | 3 |  |  |  | Below | 5.6 | Severe |
| **16** | LCA | 1 | Semi-selective | 4 | 17 | Small sinus  Commissural post  Pericardial skirt | Wire-assisted (AL2) | Perimount 23 | ACURATE S | 8 | 30 | 28 | -0.5 | Below | 6.2 | Moderate |
|  |  | 2 | Selective | 3 | 10,12 | Small sinus  Commissural post  Pericardial skirt | Wire-assisted (AL1) |  |  |  |  |  |  |  |  |  |
| **19** | LCA | 1 | Selective | 2 | 5,25 | Small sinus | Standard  (JL5) | Mitroflow 25 | ACURATE S | 6.5 | 31 | 34 | 5.9 | Below | 6.1 | Mild |

*RCA=right coronary artery, LCA=left coronary artery, SAVR=surgical aortic valve replacement, TAVR=transcatheter aortic valve replacement, SoV=sinus of Valsalva, STJ=sinotubular junction,*

**Supplementary Table 3: Overview of procedural, valvular and imaging data for each challenging guiding catheter cannulation**

|  |  |  | **CANNULATION** | | | | **VALVE** | | **PRE-PROCEDURE CT** | | | **POST-PROCEDURE CT** | | | |
| --- | --- | --- | --- | --- | --- | --- | --- | --- | --- | --- | --- | --- | --- | --- | --- |
| **Pt** | **Artery** | **Operator** | **Type** | **Attempts** | **Fluoroscopy time**  **(mins,sec)** | **Technique** | **SAVR** | **TAVR** | **Coronary height** | **SoV width** | **STJ width** | **Implant depth** | **Above or below RP** | **MTC** | **Co-Cor angle** |
| **1** | RCA | 2 | Selective | 3 | 9,30 | Wire-assisted (JL4) | Mitroflow 27 | ACURATE Size M | 5 | 36 | 32 | 9.9 | Below | 3 | Mild |
| **2** | LCA | 1 | Not feasible | 3 | >5 | - | Mitroflow 23 | ACURATE  Size S | 3.5 | 28 | 25 | 3.8 | Below | 5 | Severe |
|  | RCA* | 1 | Not feasible | 3 | >5 | - |  |  | 4 |  |  |  | Below | 4.7 | Severe |
|  |  | 2 | Not feasible | 3 | >5 | - |  |  |  |  |  |  |  |  |  |
| **3** | LCA | 1 | Non-selective | 3 | 19,11 | Wire-assisted  (AL1) | Perimount 19 | ACURATE  Size S | 3 | 31 | 28 | 3.6 | Below | 4 | Moderate |
|  | RCA | 1 | Non-selective | 1 | 3,31 | Wire-assisted (JR4) |  |  | 6 |  |  |  | Below | 6.9 | Severe |
|  |  | 2 | Selective | 3 | 23,27 | Balloon-assisted (IM) |  |  |  |  |  |  |  |  |  |
| **6** | LCA* | 1 | Semi-selective | 2 | 14,10 | Wire-assisted (AL1) | Mitroflow 25 | ACURATE  Size S | 11 | 52 | 39 | 8.4 | Above | 20 | Severe |
|  |  | 2 | Non-selective | 2 | 7,16 | Guide-extension (AL2) |  |  |  |  |  |  |  |  |  |
|  | RCA | 1 | Semi-selective | 3 | 11,52 | Wire-assisted (AL2) |  |  | 21 |  |  |  | Above | 22.5 | Severe |
| **11** | RCA* | 1 | Semi-selective | 3 | 14,1 | Wire-assisted (IM) | Hancock 23 | ACURATE  Size S | 10 | 32 | 28 | 4.8 | Above | 1.6 | Severe |
|  |  | 2 | Selective | 3 | 6,35 | Standard (AL1) |  |  |  |  |  |  |  |  |  |
| **14** | LCA | 2 | Non-selective | 2 | 6,10 | Guide-extension (MP1) | Perimount 23 | ACURATE  Size S | 5.2 | 25 | 25 | 3.2 | Below | 4.9 | Severe |
| **16** | LCA | 1 | Semi-selective | 2 | 5,22 | Wire-assisted (AL1) | Mitroflow 25 | ACURATE  Size S | 8 | 30 | 28 | -0.5 | Below | 6.2 | Moderate |
|  |  | 2 | Selective | 2 | 6,15 | Wire-assisted (EBU 3.5) |  |  |  |  |  |  |  |  |  |

|  | **Coronary height** | | | **Virtual Transcatheter-to-coronary**  **distance** | | |
| --- | --- | --- | --- | --- | --- | --- |
|  | <10mm | >10mm | p-value | <6mm | >6mm | p-value |
| **Cannulation time, mins**   - Diagnostic catheter - Guiding catheter | 2.3 (1.3-4.6)  3.2 (0.8-5.2) | 1.2 (1-2)  1.3 (0.7-2.4) | <0.05  0.22 | 2.5 (1.5-5.0)  4.1 (1.5-6.4) | 1.4 (0.5-2.4)  1.2 (0.4-2.5) | <0.01  < 0.01 |
|  |  |  |  |  |  |  |
| **Cannulation attempts**   - Diagnostic catheter - Guiding catheter | 1 (1-1.5)  1 (1-2) | 1 (1-1)  1 (1-1) | 0.52  <0.05 | 1 (1-2)  1 (1-2) | 1 (1-1)  1 (1-1) | 0.43  <0.01 |
|  |  |  |  |  |  |  |
| **Cannulation selectivity**  (Diagnostic catheter)   - Selective - Semi-selective - Non-selective | 27 (42%)  9 (14%)  4 (6%) | 24 (38%)  0 (0%)  0 (0%) | <0.05 | 16 (25%)  8 (13%)  2 (3%) | 35 (55%)  1 (2%)  2 (3%) | <0.01 |
| **Cannulation selectivity**  (Guiding catheter)   - Selective - Semi-selective - Non-selective | 22 (34%)  12 (19%)  6 (9%) | 18 (28%)  5 (8%)  1 (2%) | 0.25 | 12 (19%)  10 (16%)  4 (6%) | 28 (44%)  7 (11%)  3 (5%) | 0.09 |

**Supplementary table 4: Cannulation parameters according to pre-procedural CT imaging variable cut-offs**

**Supplementary table 5: Cannulation parameters according to post-procedural CT imaging variable cut-offs**

|  | **Implant depth** | | | **Relationship to**  **risk plane** | | | **Measured Transcatheter-to-coronary**  **distance** | | | **Coronary-commissural angle** | | |
| --- | --- | --- | --- | --- | --- | --- | --- | --- | --- | --- | --- | --- |
|  | <4mm | >4mm | p-value | Above | Below | p-value | <6mm | >6mm | p-value | <40^o^ | >40 ^o^ | p-value |
| **Cannulation time, mins**   - Diagnostic catheter - Guiding catheter | 2.5 (1.5-5.2)  3.7 (1.7-5.7) | 1.4 (1-2.5)  1.3 (0.5-3.9) | <0.05  <0.05 | 1.4 (0.8-2.4)  1.2 (0.3-2.4) | 2.2 (1.1-4.6)  3.2 (1.1-5.2) | <0.05  <0.05 | 2.5 (1.5-5.1)  4.0 (1.1-6.2) | 1.4 (0.8-2.8)  1.5 (0.5-3.9) | <0.05  <0.05 | 2.3 (1.4-4.1)  2.3 (0.5-5.2) | 1.1 (0.5-1.2)  1.3 (1.1-2.2) | <0.01  0.43 |
|  |  |  |  |  |  |  |  |  |  |  |  |  |
| **Cannulation attempts**   - Diagnostic catheter - Guiding catheter | 1 (1-2.5)  1 (1-2) | 1 (1-1)  1 (1-1) | <0.05  0.15 | 1 (1-1)  1 (1-1) | 1 (1-1.15)  1 (1-2) | 0.52  0.13 | 1 (1-2)  1 (1-3) | 1 (1-1)  1 (1-1) | 0.40  <0.05 | 1 (1-2)  1 (1-2) | 1 (1-1)  1 (1-1) | 0.13  <0.05 |
|  |  |  |  |  |  |  |  |  |  |  |  |  |
| **Cannulation selectivity**  (Diagnostic catheter)   - Selective - Semi-selective - Non-selective | 13 (20%)  7 (11%)  4 (6%) | 38 (59%)  2 (3%)  0 (0%) | <0.01 | 27 (42%)  9 (14%)  4 (6%) | 24 (38%)  0 (0%)  0 (0%) | <0.01 | 9 (14%)  7 (11%)  4 (6%) | 42 (66%)  2 (3%)  0 (0%) | <0.01 | 37 (58%)  9 (14%)  4 (6%) | 14 (22%)  0 (0%)  0 (0%) | 0.11 |
| **Cannulation selectivity**  (Guiding catheter)   - Selective - Semi-selective - Non-selective | 10 (16%)  8 (13%)  6 (9%) | 30 (47%)  9 (14%)  1 (2%) | 0.25 | 22 (34%)  12 (19%)  6 (9%) | 18 (28%)  5 (8%)  1 (2%) | 0.25 | 7 (11%)  8 (13%)  5 (8%) | 33 (52%)  9 (14%)  2 (3%) | <0.01 | 27 (42%)  16 (25%)  7 (11%) | 13 (20%)  1 (2%)  0 (0%) | <0.01 |
