# Supplementary figures and images for: Coronary access following ACURATE neo implantation for transcatheter aortic valve-in-valve implantation: Ex vivo analysis in patient-specific anatomies
Source: Front Cardiovasc Med. 2022 Sep 14;9:902564. doi: 10.3389/fcvm.2022.902564 (PMC9515364; doi:10.3389/fcvm.2022.902564)

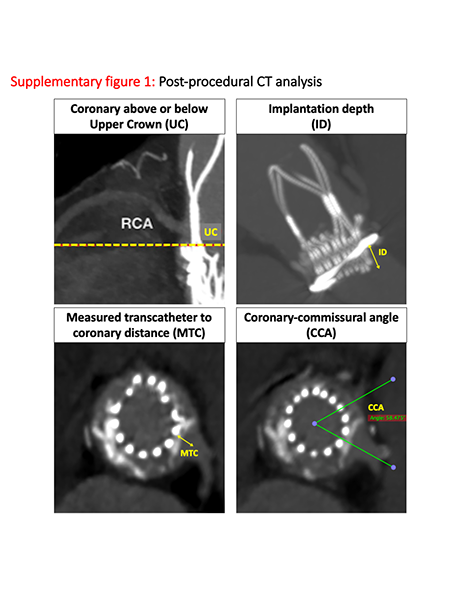

Supplement: Supplementary Figure 1 — Post-procedural CT analysis. For each coronary ostium in the cohort, post-procedural CT analysis of implantation depth, relationship to upper crown, gap between valve frame and ostium and coronary-commissural angle was performed. [file Image_1.tiff]

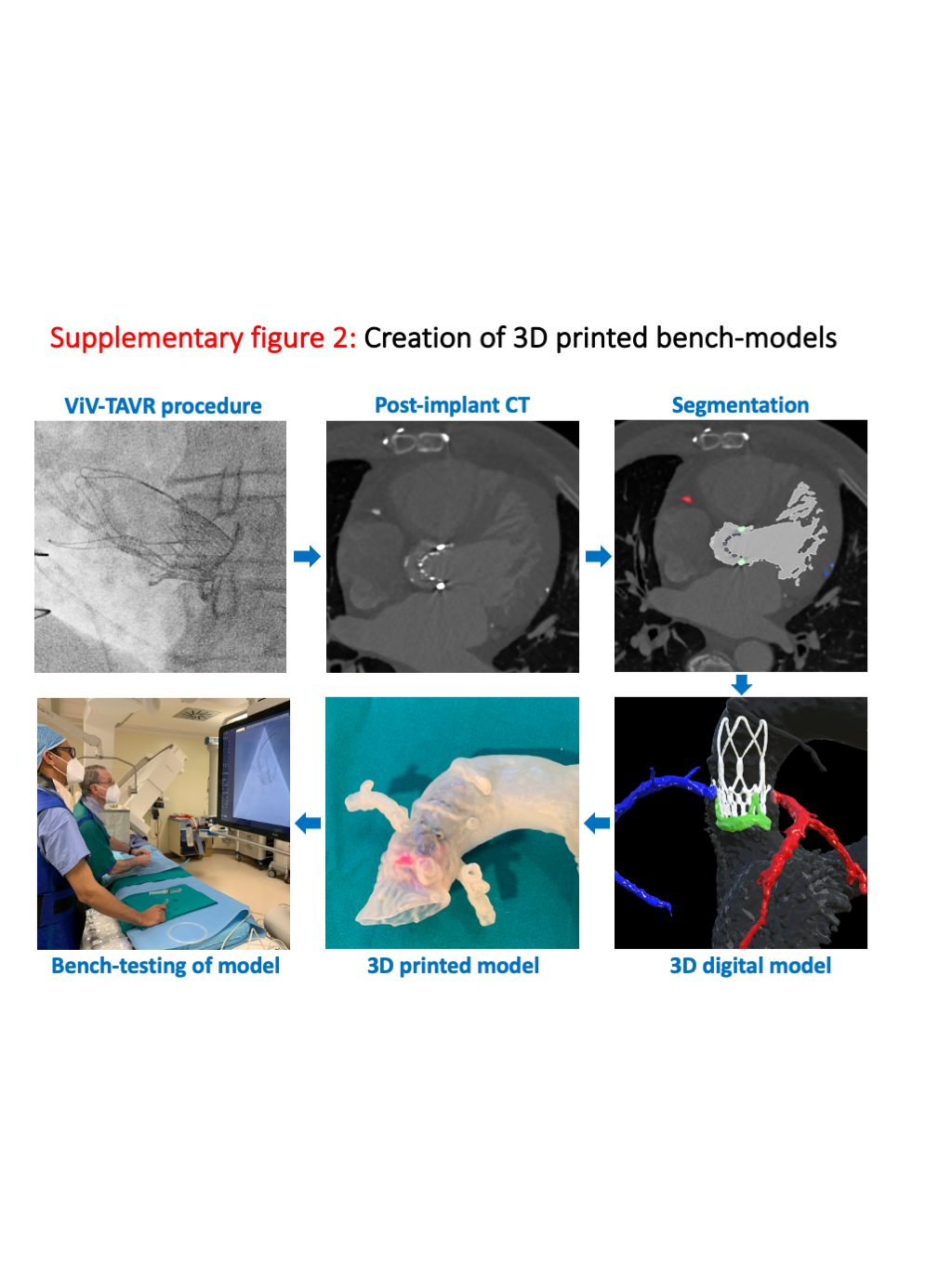

Supplement: Supplementary Figure 2 — Creation of 3D-printed model. Study flowchart demonstrating how post-procedural CT was segmented and processed to print the 3D models, which were then assembled for bench-testing under real catheterization laboratory conditions. [file Image_2.tiff]

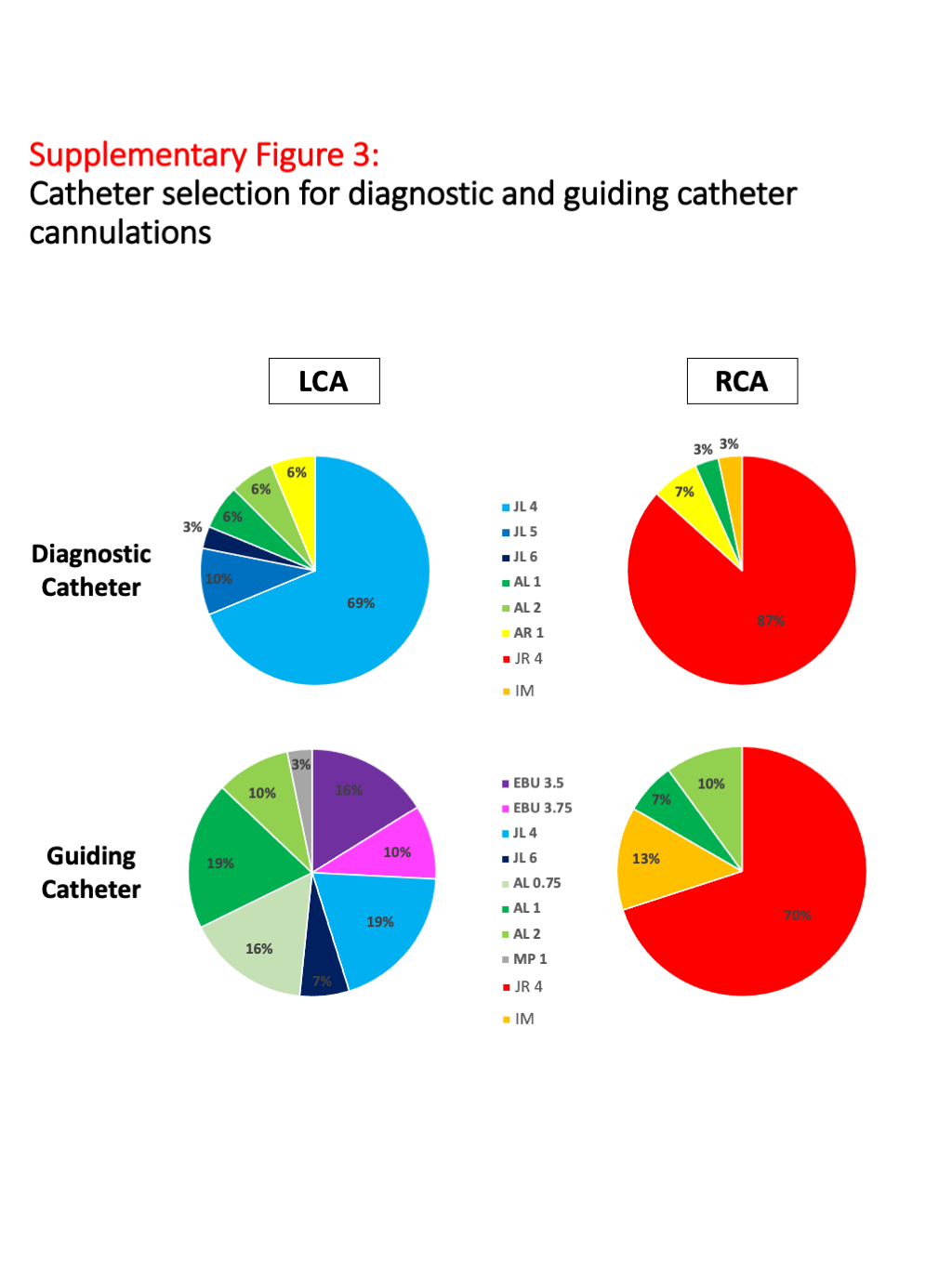

Supplement: Supplementary Figure 3 — Diagnostic and guiding catheters selected for cannulation. The Judkins Right 4 catheter was the most frequently selected catheter for the RCA whilst a greater range of catheters diagnostic and particularly guiding catheters were selected for cannulating the LCA. [file Image_3.tiff]

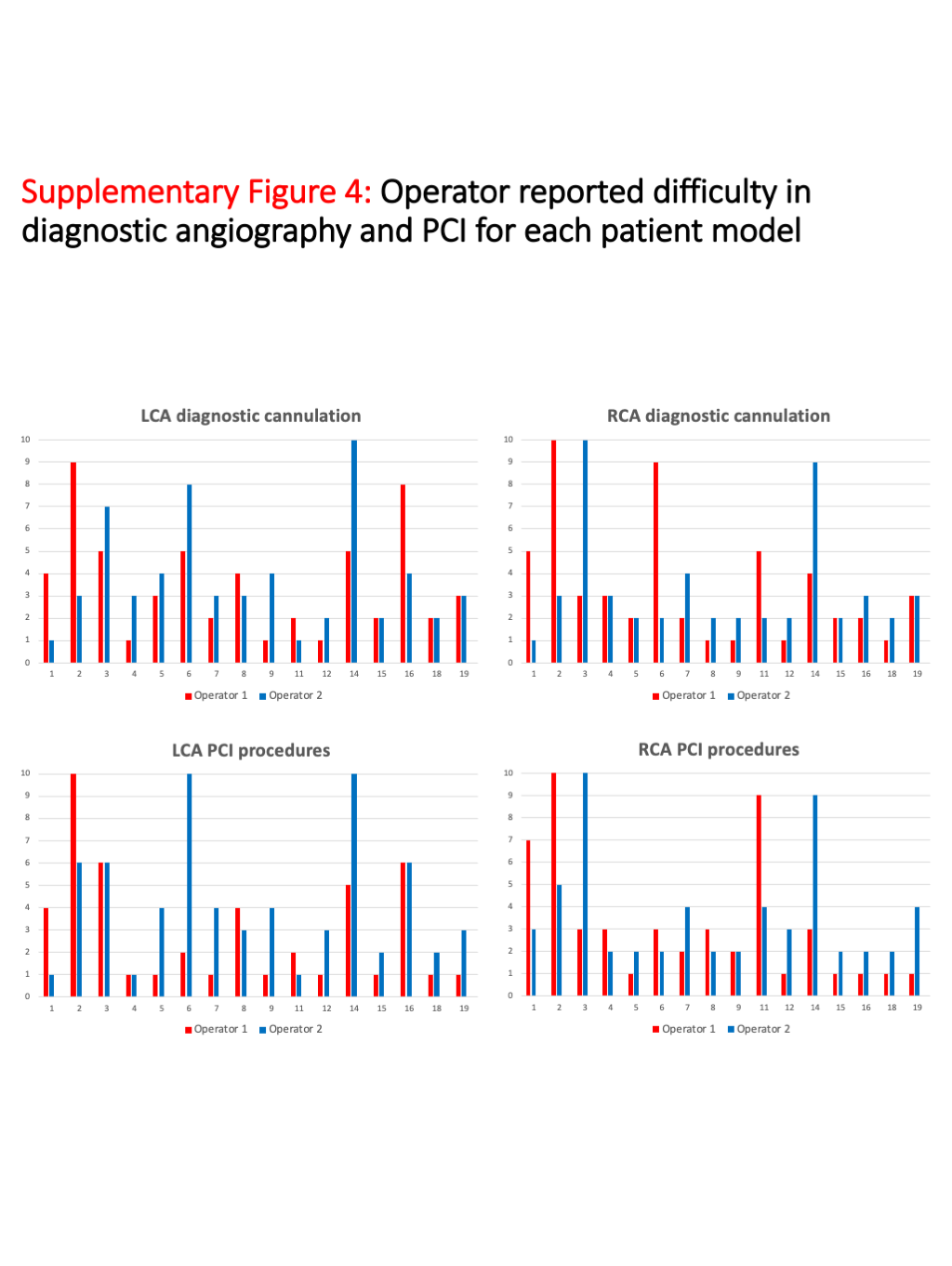

Supplement: Supplementary Figure 4 — Operator reported difficulty in diagnostic and PCI cannulation for each patient model. The two operators were asked to report their difficulty in completing the diagnostic and PCI cannulations for each ostium. Responses were recorded on a scale of 1-10 (1 = extremely easy). [file Image_4.tiff]

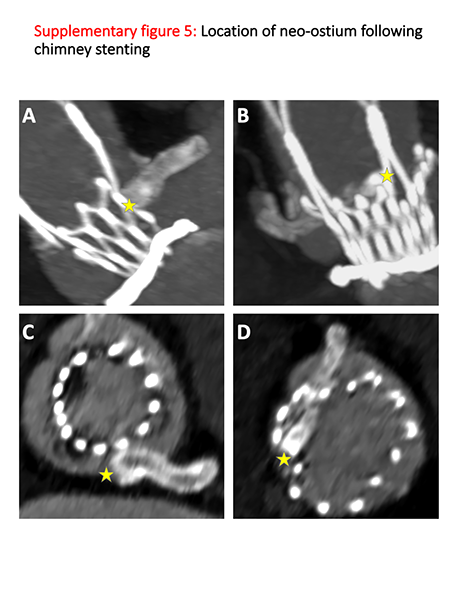

Supplement: Supplementary Figure 5 — Location of neo-ostium following chimney stenting. Case example of an 84-year old female who underwent ACURATE neo implantation to treat a degenerated Mitroflow prosthesis. Due to high-risk for coronary obstruction, coronary protection of the LCA (A,C) and RCA (B,D) using the “chimney” technique was performed. The yellow start denotes the location of the neo-stent which was un-feasible to cannulate. [file Image_5.tiff]
